# Processing Deficits of Motion of Contrast-Modulated Gratings in Anisometropic Amblyopia

**DOI:** 10.1371/journal.pone.0113400

**Published:** 2014-11-19

**Authors:** Yong Tang, Caiyuan Liu, Zhongjian Liu, Xiaopeng Hu, Yong-Qiang Yu, Yifeng Zhou

**Affiliations:** 1 CAS Key Laboratory of Brain Function and Diseases, and School of Life Sciences, University of Science and Technology of China, Hefei, Anhui, People's Republic of China; 2 Research and Treatment Center of Amblyopia and Strabismus, University of Science and Technology of China, Hefei, Anhui, People's Republic of China; 3 Department of Radiology, The First Affiliated Hospital of Anhui Medical University, Hefei, Anhui, People's Republic of China; 4 State Key Laboratory of Brain and Cognitive Science, Institute of Biophysics, Chinese Academy of Science, Beijing, People's Republic of China; University of Waterloo, Canada

## Abstract

Several studies have indicated substantial processing deficits for static second-order stimuli in amblyopia. However, less is known about the perception of second-order moving gratings. To investigate this issue, we measured the contrast sensitivity for second-order (contrast-modulated) moving gratings in seven anisometropic amblyopes and ten normal controls. The measurements were performed with non-equated carriers and a series of equated carriers. For comparison, the sensitivity for first-order motion and static second-order stimuli was also measured. Most of the amblyopic eyes (AEs) showed reduced sensitivity for second-order moving gratings relative to their non-amblyopic eyes (NAEs) and the dominant eyes (CEs) of normal control subjects, even when the detectability of the noise carriers was carefully controlled, suggesting substantial processing deficits of motion of contrast-modulated gratings in anisometropic amblyopia. In contrast, the non-amblyopic eyes of the anisometropic amblyopes were relatively spared. As a group, NAEs showed statistically comparable performance to CEs. We also found that contrast sensitivity for static second-order stimuli was strongly impaired in AEs and part of the NAEs of anisometropic amblyopes, consistent with previous studies. In addition, some amblyopes showed impaired performance in perception of static second-order stimuli but not in that of second-order moving gratings. These results may suggest a dissociation between the processing of static and moving second-order gratings in anisometropic amblyopia.

## Introduction

Amblyopia is a developmental visual disorder, which may be a consequence of sensory impediment to visual development, such as strabismus (ocular misalignment) or anisometropia (unequal refractive error), occurring early in life [1]. It is often characterized by reduced spatial vision [2]–[5], which cannot be significantly improved by refractive correction, and is believed to be a cortical disorder [6]–[8].

It has been indicated that alterations in response properties of neurons in V1, including reduced spatial resolution [9] and contrast sensitivity [10], [11], as well as a loss of proportion of cells driven by amblyopic eyes [12], [13] underlie the visual deficits in patients with amblyopia. In addition to this, many studies on human amblyopia [14]–[20] and animals with experimental amblyopia [13], [21], [22] have suggested that functions of extra-striate cortex may also be affected by amblyopia. Now, the cortical loci for processing deficits in amblyopia remain an open question.

Investigations of the perception of first- and second-order stimuli in amblyopia may be helpful to the understanding of the cortical deficits in amblyopia. First-order stimuli are defined by modulation of luminance, while second-order stimuli are defined by changes in image features, such as contrast or texture [23]. It is generally recognized that the first-order processing mechanism involves linear neurons in area V1 that detect spatial luminance variations across their receptive fields, while the processing of second-order stimuli involves three successive stages: a first-stage linear filter which is identical to first-order processing and can be conducted in area V1, a pointwise nonlinearity such as rectification, and a second-stage linear filter which has been associated with computations at extra-striate cortex [24], [25].

A large amount of studies [2], [3], [5], [26]–[28] have indicated a significant first-order loss in amblyopia, consistent with the physiological evidence described above that functions of striate cortex are affected by amblyopia. Similarly, some studies [29]–[34] have indicated substantial deficits in perception of second-order stimuli in amblyopia. By measuring the detection threshold of 5 amblyopes, Wong and his colleagues found that four amblyopic eyes and two non-amblyopic eyes showed second-order loss relative to the control eyes [29]. Additionally, they found that the second-order loss was greater than the first-order loss at the carrier spatial frequency, i.e. first-order input to second-order processing systems. Similar results were also obtained by Mansouri and his colleagues in a coarse second-order orientation (vertical vs. horizontal) discrimination task [30]. In two other studies [31], [32], Simmers and her colleagues found substantial deficits in processing of global second-order motion in amblyopes by using a specific method, which could exclude the influence of the deficits in spatial contrast sensitivity, i.e. first-order processing deficits, on motion perception. These findings were further supported by Aaen-Stockdale and his colleagues, who found significant processing deficits of translational, radial and rotational motion in amblyopia with the use of second-order random dots [33]. Simmers and her colleagues also measured the contrast sensitivity for first-order and second-order motion over a five-octave range of spatial and temporal frequencies in three patients with strabismus amblyopia [34]. They found that compared to normal controls, amblyopes were not only impaired in the processing of first-order motion, but overall they exhibited both higher thresholds and a much narrower window of visibility to second-order motion. These findings improved our understanding of the visual problems in amblyopes, and suggested that there are substantial second-order processing deficits, part, if not most, of which may originate from extra-striate cortex, in amblyopia.

Most of the studies described above have focused on the perception of static second-order stimuli [29], [30] and the global processing for second-order stimuli [30]–[33]. In contrast, less is known about the perception of second-order moving gratings in amblyopia. We therefore evaluated this issue using a motion direction discrimination task in the present study. Contrast sensitivity for second-order moving gratings was measured and compared between the AEs and NAEs of amblyopic subjects and CEs of normal control subjects. To exclude the possible influence of low-level processing deficits, i.e. first-order deficits, on the perception of second-order motion, both non-equated carriers (with a full contrast of 1.0) and a series of equated carriers were used. The setting of the equated carriers was similar to that by Wong and his colleagues [29]. And for comparison, the sensitivity for first-order motion and static second-order stimuli were also measured.

## Methods

### Subjects

Seven anisometropic amblyopes and ten normal controls with appropriate optical correction participated in this experiment. The visual characteristics of all amblyopes were given in Table 1. The average age of the amblyopes was 23.7±0.3 years, and that of the normal controls was 24.6±2.1 years. All observers were naïve to the purpose of experiment.

**Table 1:** Visual Characteristics of Amblyopic subjects.

Subject	Sex	Age	Type	Optical Correction	Visual acuity (MAR)
S1	M	23	A	AE +2.00DS/+3.00DC×42	2.0
				NAE −0.50DS×165	1.0
S2	M	25	A	AE +6.00/+0.75×42	2.5
				NAE −0.75/−0.50×161	0.8
S3	M	23	A	AE +2.00DS/+2.50DC×95	4.0
				NAE −3.75DS/−0.75DC×10	1.0
S4	F	24	A	AE +2.50DS/1.50DC×85	2.5
				NAE plano	0.8
S5	M	24	A	AE +1.00DS/+1.50DC×95	2.0
				NAE Plano	1.0
S6	F	23	A	AE +4.00DS	6.7
				NAE Plano	1.0
S7	M	24	A	AE +4.00DS/+1.00DC×85	8.3
				NAE Plano	1.0

F, female; M, male; A, anisometropic amblyopia; AE, amblyopic eyes; NAE, non-amblyopic eyes; MAR, minimum angles of resolution.

### Ethics statement

This research has been approved by the ethics committee of the University of Science and Technology of China (We cannot provide the permit number because our institution regulations do not require such numbers), and was performed in accordance with the ethical standards laid down in the 1964 Declaration of Helsinki. The written informed consent was obtained from all participants before participation.

### Apparatus

All stimuli were generated in real time using programs in Matlab 6.5 with Psychtoolbox (version 2.50) extensions [35], [36]. The computer was a P4 PC, with an ATI 7500 video card and a 17-inch Sony G220 monitor. A special circuit was used to combine two 8-bit output channels of the video card to produce 14 bits of gray levels [37]. Luminance calibration was performed by using a psychophysical procedure in combination with a photometer (UDT 161) [37], [38]. The mean background luminance was set to 50 cd/m^2^. When measuring the spatial contrast sensitivity function, the screen resolution was 1600×1200 pixels with a frame rate of 75 Hz, and the eye-screen distance was 228 cm. When measuring the contrast sensitivity for first- and second-order motion, the screen resolution was 640×480 pixels with a frame rate of 160 Hz, and the viewing distance was 114 cm. A chin rest was used to fix head position. All viewing was monocular in a dimly lit room.

### Stimuli

The stimuli used for spatial contrast sensitivity measurements were sine-wave gratings with different spatial frequencies, which were 0.5, 1, 2, 4, 8, 16 and 24 c/d for amblyopic eyes, and were 0.5, 1, 2, 4, 8, 16, 24 and 32 c/d for non-amblyopic eyes. These stimuli subtended 3.8°×3.8°, and were presented at the center of the screen.

**Figure 1:**
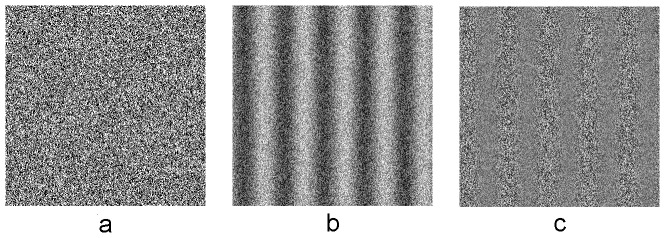
Illustration of stimuli used in this experiment. (a) noise carrier, (b) first-order stimulus, (c) second-order stimulus. All stimuli are presented at screen center, subtended 2.51°×2.51°.

The noise carrier (Figure 1a) was a 1-bit, spatially 2-d, static noise pattern generated by assigning individual (single) screen pixels (0.025 degree of visual angle) to be either ‘white’ or ‘black’ with equal probability to ensure that there was no spatial variation in luminance within individual noise elements. A new stochastic noise sample was used for each trial. Note that the use of this noise size would not significantly introduce luminance artifacts [39], [40].

The first-order motion stimulus (Figure 1b) was a noise carrier whose luminance was modulated by a sinusoidal grating, while the second-order motion stimulus (Figure 1c) was a noise carrier whose contrast was modulated by a sinusoidal grating. The luminance profile at point (x, y) of the first- (Equation 1) and second-order (Equation 2) stimuli are defined as:
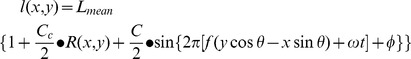
(1)

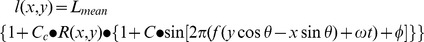
(2)where 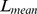 is the background luminance of the display; 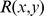 is the carrier; 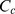 is the contrast of the carrier (The Michelson contrast is 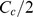 for first-order motion); 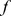 is the spatial frequency of the envelope grating (here set to 1 cycle/degree); 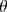 represents the orientation of the envelope grating (here set to 90°); 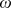 is the temporal frequency of the envelope grating (1.0, 2.0, 4.0, 8.0, 16.0, and 30.0 Hz for first-order stimuli; 1.0, 2.0, 4.0, 8.0, and 16.0 Hz for second-order stimuli; 0 Hz for static stimuli); 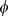 is the (random) initial spatial phase; and 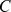 is the contrast of the envelope, i.e. first-order or second-order modulation depth. These stimuli subtended 2.51°×2.51°, and were presented at the center of the screen. The edges of the square window in which these stimuli were displayed were abrupt.

### Experimental design and procedure

With a stimulus detection task, the spatial contrast sensitivity function was first measured in amblyopic eyes (AE) and non-amblyopic eyes (NAE) of amblyopic subjects and dominant eyes (CE) of normal control subjects (The results of spatial contrast sensitivity function were not shown here because they were very similar to those described in previous studies [28], [41]). A carrier detection task was then used to measure the contrast threshold for the noise carriers. Subsequently, a static stimuli orientation discrimination task and a motion direction discrimination task were applied. The order of two discrimination tasks was counter-balanced between subjects. For each eye, the discrimination threshold was first measured with non-equated carriers, i.e. the noise carrier contrast 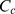 was set to 1.0. Following this, it was measured with a number of carrier contrast levels in order to equate carrier visibility, i.e. with equated carriers. Levels were specified in carrier contrast threshold units (CCTU), which were multiples of carrier contrast threshold measured in the carrier detection task.

A two-alternative-forced-choice design was used in this experiment. In the noise carrier detection task, subjects were asked to detect the presence of the stimulus, which was presented in one of two successive presentation intervals each lasting 250 ms and separated by a 500 ms inter-stimulus interval (ISI). In the stimulus discrimination task, subjects were required to indicate the orientation of the static second-order stimulus, horizontal or vertical, and the direction of first-order or second-order motion, leftwards or rightwards. The duration of all stimuli was 250 ms with 25 ms linear ramps in the beginning and the end.

Contrast thresholds were measured using a two-down one-up staircase procedure [42], which decreased the signal contrast by 10% (*c_t+1_* = 0.90*c_t_*) following every two consecutive correct responses and increased the signal contrast by 10% (*c_t+1_* = 1.10*c_t_*) after every incorrect answer, converging on 70.7% correct. The starting contrast of the staircase was determined by a prior study. Each staircase ran through 100 trials, usually generating about 20 reversals. When measuring the spatial contrast sensitivity and the contrast thresholds for first- and second-order motion, trials for the staircases associated with each spatial or temporal frequency condition were intermixed randomly. Contrast sensitivity (reciprocal of contrast threshold) was used for data analysis.

All trials were initiated by the subjects. In each trial, the stimulus was preceded by a short beep. Then the subject indicated her/his decision with a keyboard button press. No feedback was provided. Before the measure, each subject received a short practice session.

### Statistical analysis

Between-subject ANOVA and *t*-test were used to compare data in the amblyopic eyes of the amblyopic subjects and the dominant eyes of the control subjects. The same statistical tests were also used to compare data in the non-amblyopic eyes of the amblyopic subjects and the dominant eyes of the control subjects. Within-subject ANOVA and *t*-test were used to compare data in the amblyopic and the non-amblyopic eyes of the amblyopic subjects. All data were expressed as Mean±SEM.

## Results

### First-order motion

**Figure 2:**
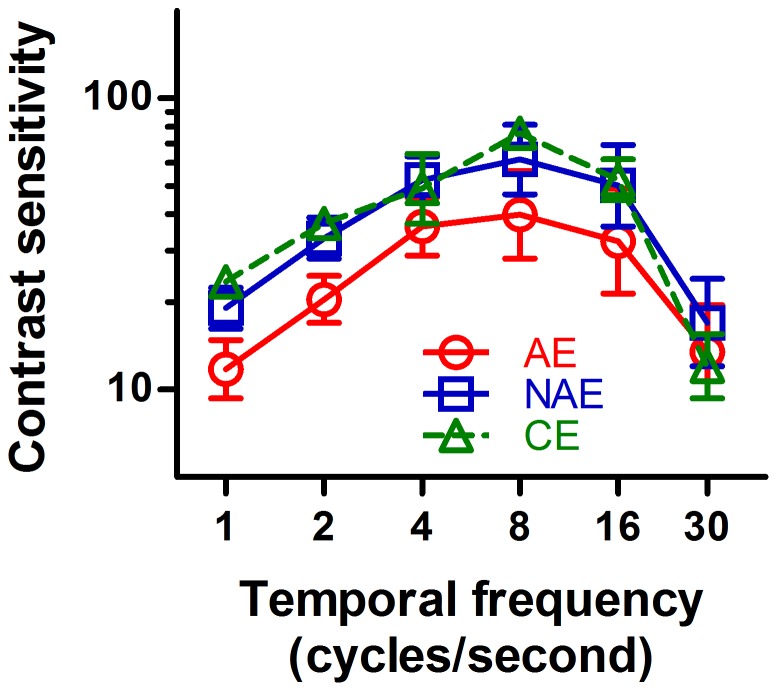
Average contrast sensitivity for the identification of the direction of first-order motion for amblyopic eyes (AE, Circles), non-amblyopic eyes (NAE, Squares), and control eyes (CE, Triangles). Error bars represent one standard error of the mean.

The average contrast sensitivity for first-order motion is plotted as a function of the temporal frequency for AEs, NAEs and CEs in Figure 2. As shown in the figure, all curves exhibit inverted U-shaped patterns, and the peaks of all curves occur around 8 Hz.

In this condition, although the sensitivity function of the AEs is slightly lower than those of NAEs and CEs, the differences were not statistically significant (AE vs. NAE, within-subject ANOVA, *F*_1,6_ = 2.948, *p* = 0.137; AE vs. CE, between-subject ANOVA, *F*_1,15_ = 2.600, *p* = 0.128). And there were no significant differences in the sensitivity functions between the NAEs and the CEs (between-subject ANOVA, *F*_1,15_ = 0.015, *p* = 0.904). In all cases, the two-way interactions were not statistically significant (all *p*'s>0.05). These results indicated that the contrast sensitivity for sine-wave moving gratings, i.e. first-order motion, at the spatial frequency of 1 c/d was relatively normal in these amblyopic subjects.

### Sensitivity for the carriers

**Figure 3:**
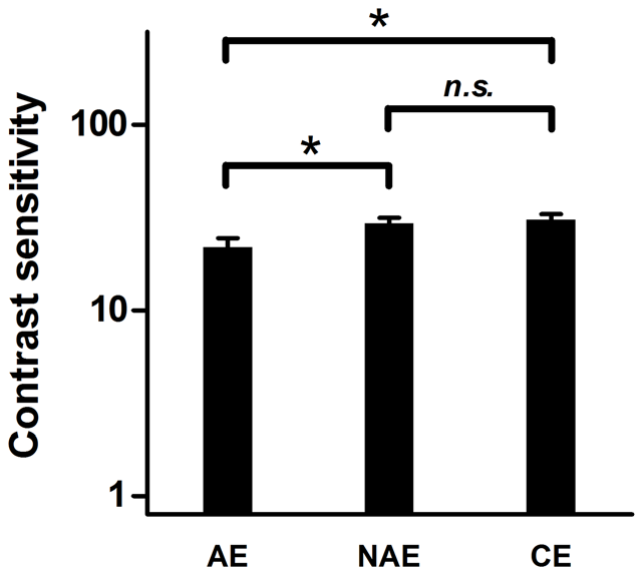
Averaged contrast sensitivity for carriers in amblyopic eyes, non-amblyopic eyes and control eyes. AE, amblyopic eyes; NAE, non-amblyopic eyes; CE, control eyes. *, statistically significant (*p*<0.05); *n.s.*, non-significant (*p*>0.05). Error bars represent one standard error of the mean.

To evaluate the first-order processing deficits in the perception of second-order motion in anisometropic amblyopia, the detection threshold for the noise carriers was measured, with the results shown in Figure 3. For the noise carrier, the average contrast sensitivity was 22.77±2.77, 30.10±1.90 and 31.56±2.09 for AEs, FEs, and CEs, respectively. There was no significant difference between the sensitivity of the NAEs and CEs (between-subject *t*-test, *t*_15_ = 0.492, *p* = 0.630), whereas the sensitivity of the AEs were significantly lower than those of the NAEs (within-subject *t*-test, *t*_6_ = 4.220, *p* = 0.006) and those of the CEs (between-subject t-test, *t*_15_ = 2.585, *p* = 0.021). These results indicated substantial deficits in perception of the noise carriers in AEs but not in NAEs, consistent with the previous result [30].

### Second-order motion

**Figure 4:**
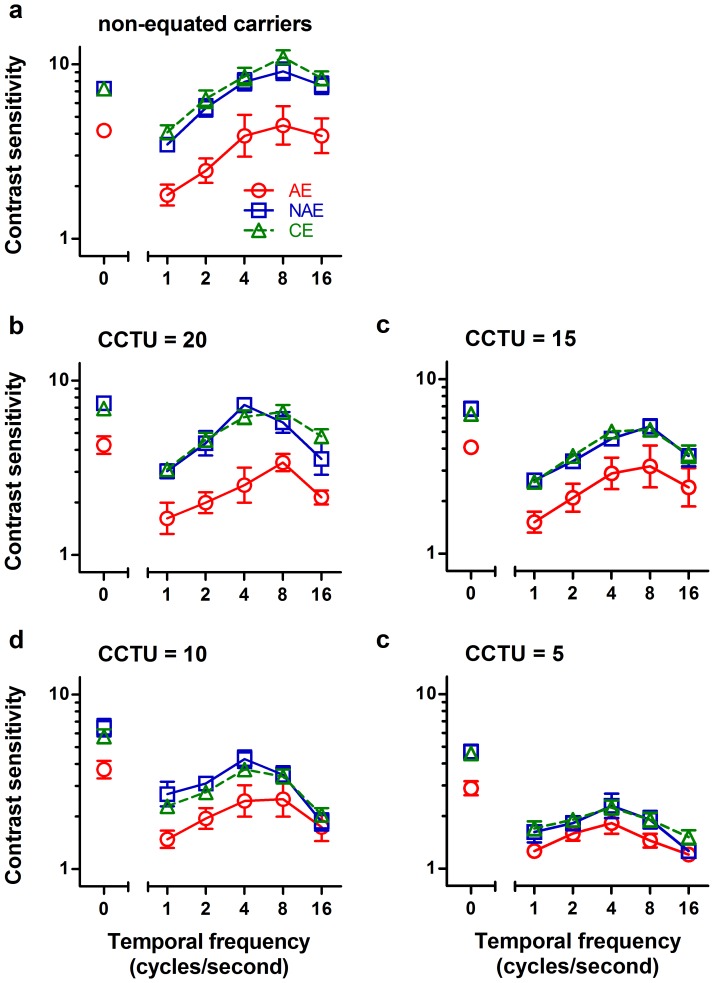
Average contrast sensitivity for second-order motion and static second-order stimuli for amblyopic eyes (AE, Circles), non-amblyopic eyes (NAE, Squares), and control eyes (CE, Triangles). Error bars represent one standard error of the mean.

**Figure 5:**
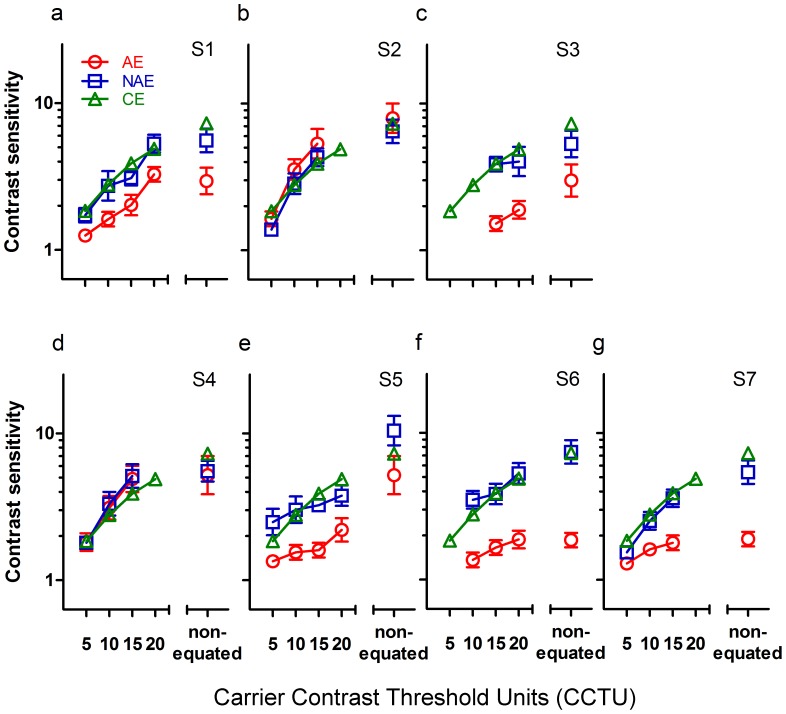
Contrast sensitivity for second-order motion at all carrier contrast levels for each amblyopic subject. The data points for control eyes (CE, Triangles) were averaged across subjects and temporal frequencies. While the data points for amblyopic eyes (AE, Circles) and non-amblyopic eyes (NAE, Squares) were only averaged across temporal frequencies. Note that for some subjects (S3 and S6) the sensitivity for second-order motion could not be measured at some low carrier contrast levels because of the floor effect. And for some other subjects (S2, S4 and S7) the data could not be measured at the highest carrier contrast levels because in this condition the expected contrast of the noise carriers is larger than 1. Error bars represent one standard error of the mean.

Data for second-order motion with non-equated and a series of equated carriers are shown in Figure 4. For comparison, the sensitivity for static second-order stimuli was also shown. From this figure, it is clear that all eyes exhibited a better performance at a higher CCTU, both for second-order motion and for static second-order stimuli. Note that there are different numbers of participants in each condition tested, which can be clearly seen in Table 2 and Figure 5.

**Table 2:** Comparisons of contrast sensitivity for static second-order gratings.

		*df*	*t*	*p*
AE vs. CE*	non-equated	15	6.986	<0.001
	CCTU20	12	4.840	<0.001
	CCTU15	15	4.674	<0.001
	CCTU10	14	3.128	0.007
	CCTU5	13	3.674	0.003
AE vs. NAE#	non-equated	6	6.556	0.001
	CCTU20	3	0.531	0.012
	CCTU15	6	7.027	<0.001
	CCTU10	5	4.011	0.010
	CCTU5	4	3.577	0.023

AE, amblyopic eyes; NAE, non-amblyopic eyes; CE, dominant eyes of control subjects.

*, between-subject *t*-test;

# , within-subject *t*-test.

As expected, the AEs showed significantly reduced sensitivity to static second-order stimuli relative to the CEs and NAEs at all carrier contrast levels, and there were no significant differences in sensitivity between the NAEs and CEs (Table 2). These findings suggest substantial processing deficits in static second-order stimuli, consistent with the previous studies [29], [30].

Similar to those for first-order motion, all curves for second-order motion show inverted U-shaped patterns, and most of them peak at around 8 Hz. In all carrier contrast levels, sensitivity for second-order motion seems to be comparable between the NAEs and CEs (between-subject ANOVA, non-equated, *F*_1,15_ = 1.200, *p* = 0.291; CCTU20, *F*_1,12_ = 0.354, *p* = 0.563; CCTU15, *F*_1,15_ = 0.038, *p* = 0.847; CCTU10, *F*_1,14_ = 0.549, *p* = 0.471; CCTU5, *F*_1,13_ = 0.176, *p* = 0.682). In contrast, AEs showed significantly reduced performance relative to CEs (between-subject ANOVA, non-equated, *F*_1,15_ = 19.9820, *p*<0.001; CCTU20, *F*_1,12_ = 33.199, *p*<0.001; CCTU15, *F*_1,15_ = 7.762, *p* = 0.014; CCTU10, *F*_1,14_ = 5.084, *p* = 0.041) except for the lowest carrier contrast levels (CCTU5, *F*_1,13_ = 4.263, *p* = 0.059). Similarly, significant differences were also found between the AEs and the NAEs at high carrier contrast levels (within-subject ANOVA, non-equated, *F*_1,6_ = 14.882, *p* = 0.008; CCTU20, *F*_1,3_ = 31.067, *p* = 0.011; CCTU15, *F*_1,6_ = 9.152, *p* = 0.023) but not at low levels (CCTU10, *F*_1,5_ = 5.520, *p* = 0.066; CCTU5, *F*_1,4_ = 1.877, *p* = 0.243).

It seems that the performance gap between high and low carrier contrast levels described above was mainly caused by the performance variance between these amblyopic subjects, which can be clearly seen in Figure 5. In this figure, the data points for control eyes were averaged across 10 subjects and 5 temporal frequencies. While the data points for amblyopic eyes (AE, Circles) and non-amblyopic eyes (NAE, Squares) were only averaged across temporal frequencies. For most of the amblyopic subjects (S1, S3, S5, S6 and S7), NAEs and CEs showed much better performance than AEs in all conditions (in all cases, *p*<0.05). Whereas for S2 and S4, AEs had similar sensitivity to NAEs (within-subject ANOVA, S2, *F*_1,4_ = 3.037, *p* = 0.156; S4, *F*_1,4_ = 0.493, *p* = 0.521), and even to the CEs (between-subject ANOVA,S2, *F*_1,53_ = 0.953, *p* = 0.333; S4, *F*_1,53_ = 0.001, *p* = 0.990). In addition, because of the floor effect, the sensitivity for second-order motion could not be measured at low carrier contrast levels, such as CCTU5 and CCTU10, for amblyopic subjects (S3 and S6) with worse performance. As a result, the group-averaged performance included less data from these subjects at low carrier contrast levels, and therefore biased to “normal performance” in these conditions.

### Contrast sensitivity ratios

**Figure 6:**
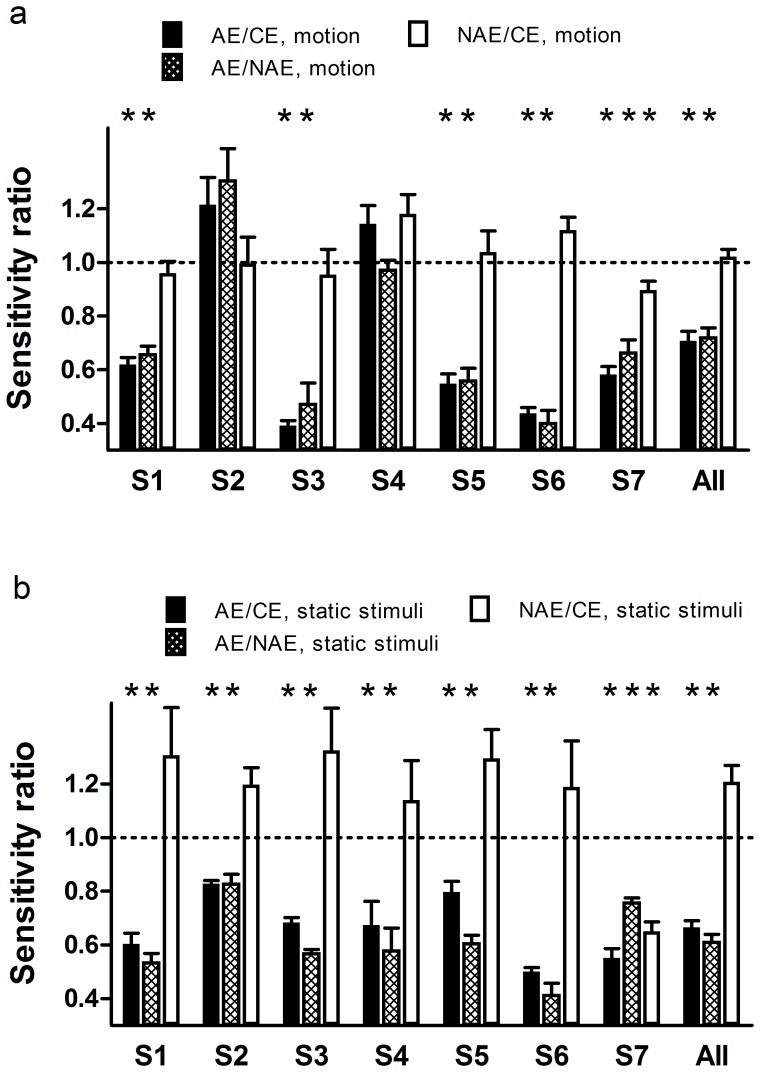
Averaged sensitivity ratios between the amblyopic eyes (AE), non-amblyopic eyes (NAE), and control eyes (CE) for second-order motion (a) and static second-order stimuli (b). Dash line, the value (1) used in *t*-test. *, statistically different from 1 (*p*<0.05) and a mean value lower than 1.

To get a more clear sense of the deficits in perception of second-order motion in anisometropic amblyopia, the sensitivity ratios between the AEs, NAEs and CEs were calculated for each amblyopic subject (Figure 6a). The ratios were averaged across 4 carrier contrast levels (CCTU5, CCTU10, CCTU15 and CCTU20) and 5 temporal frequencies. Note that data from measures with non-equated carriers were not included. From Figure 6a, it is clear that most of the AE/CE and AE/NAE ratios (5/7, except S2 and S4) were significantly (all *p*'s<0.05) lower than 1, and that most of the NAE/CE ratios (6/7, except S7) were statistically (all *p*'s>0.1) comparable to 1. As a group, the AE/CE and AE/NAE ratios were 0.71±0.03 and 0.72±0.03, respectively, significantly different from 1 (two-tailed one-sample *t*-test, AE/CE ratios, *t*_109_ = −8.561, *p*<0.001; AE/NAE ratios, *t*_109_ = −8.077, *p*<0.001), and the NAE/CE ratio was 1.02±0.03, statistically comparable to 1 (two-tailed one-sample *t*-test, *t*_109_ = 0.828, *p* = 0.409). These results suggest that the processing of second-order motion is substantially impaired in AEs of anisometropic amblyopes. In contrast, most of the NAEs are relatively spared.

For comparison, the sensitivity ratios between the AEs, NAEs and CEs were also calculated for static second-order stimuli, which were shown in Figure 6b. The AE/CE and AE/NAE ratios varied between subjects, from 0.41 (S6, AE/NAE) to 0.83 (S2, AE/CE and AE/NAE), and were all lower than 1 in statistics (two-tailed one-sample *t*-test, all *p*'s<0.05). As to NAE/CE ratios, most were slightly larger than 1, and only those of S7 were not (S7, 0.61, 0.62 and 0.72 at CCTU15, CCTU10 and CCTU5, respectively; two-tailed one-sample *t*-test, *t*_2_ = −10.000, *p* = 0.01). All these findings suggest that the perception of static second-order stimuli was strongly impaired in AEs and part of the NAEs of anisometropic amblyopes, consistent with the previous study [29].

It should be noted that, in Figure 6, the AEs of S2 and S4 had “normal” sensitivity for second-order motion (S2, AE/CE ratios = 1.21±0.10, AE/NAE ratios = 1.30±0.12; S4, AE/CE ratios = 1.14±0.07, AE/NAE ratios = 1.18±0.07). In contrast, this is not the case for static second-order stimuli. In most cases, their ratios were significantly lower than 1 (S2, AE/CE ratios = 0.83±0.01, two-tailed one-sample *t*-test, *t*_2_ = −15.248, *p* = 0.004; AE/NAE: 0.83±0.03, two-tailed one-sample *t*-test, *t*_2_ = −5.076, *p* = 0.037; S4, AE/CE: 0.58±0.08, two-tailed one-sample *t*-test, *t*_3_ = −5.136, *p* = 0.036; AE/NAE: 0.67±0.09, two-tailed one-sample *t*-test, *t*_2_ = −3.696, *p* = 0.066), indicating that the processing of static second-order stimuli was substantially impaired in their AEs. These results might suggest a dissociation between the processing of static second-order stimuli and second-order motion in anisometropic amblyopia.

## Discussion

In the present study, we found poor contrast sensitivity for second-order moving gratings in amblyopic eyes of anisometropic amblyopes, even after eliminating the influence of low-level processing deficits. In contrast, the non-amblyopic eyes were relatively spared. We also found that contrast sensitivity for static second-order stimuli was strongly impaired in AEs and part of the NAEs of anisometropic amblyopes, consistent with previous studies. Moreover, some amblyopes showed impaired performance in perception of static second-order stimuli but not in that of second-order moving gratings. All these results suggest that the core mechanisms for second-order processing are strongly affected by amblyopia.

### Sensitivity for first- and second-order motion

A number of previous studies have measured the contrast sensitivity for first- and/or second-order motion in normal adults [34], [43]–[46] and amblyopes [34], [46]. The sensitivity functions shown in the present study were similar to those found previously, except for two minor points.

First, the contrast sensitivity for first-order motion in this study was a little lower than that in previous studies. With monocular viewing and a slightly larger stimulus size (3.1 degrees), our previous study [46] has shown that, for normal controls, the peak value of the sensitivity function for moving gratings at 1 c/d is larger than 100. However, the first-order stimulus was only a sine-wave grating in that study, whereas the stimulus was composed of a sine-wave grating and a random noise carrier in the present study. We emphasize that an added noise carrier could greatly reduce the contrast sensitivity for first-order stimuli, no matter static or moving [45]. Additionally, the stimulus duration was 250 ms in the present study, which is much smaller than those (>600 ms) used in some other previous studies [34], [43], [44]. On this basis, it is not surprising that the contrast sensitivity for first-order motion in the present study seems to be a little lower than previously found.

Second, the peak of the sensitivity function for second-order motion occurs at 8 Hz in some conditions of the present study, which was a little higher than previously found in normal adults [34], [43], [44]. This difference may be associated with the refresh rate of the monitor used in the experiment. It was 160 Hz in the present study, much higher than previously used (75 Hz). A high refresh rate may provide more precise information about the motion at high temporal frequencies, such as 8 Hz, which may therefore improve the sensitivity at high temporal frequencies and change the peak location. This peak difference may also be associated with the contrast of the noise carriers. In previous studies [34], [43], [44], the carrier contrasts were 0.15–0.30. While in the present study, a much wider range was employed, from about 0.15 (CCTU5) to 1 (non-equated). Note that in conditions with high carrier contrasts (non-equated carriers, CCTU15 and part of CCTU20) in the present study, the peak occurs at around 8 Hz, whereas in conditions with low contrasts (CCTU10 and CCTU5), the peak moved to lower temporal frequencies, which were similar to previously results. This idea was also supported by one previous study [47] which used a carrier contrast of 1 and found similar sensitivity in normal adults for contrast-modulated motion at 3.75 Hz and 7.5 Hz.

### Second-order processing deficits in amblyopia

As described in the Introduction, the previous studies [29], [30], [34] have investigated the processing of second-order gratings with various tasks and several types of amblyopia. Wong and his colleagues employed a static stimulus detection task and patients with both anisometropic and strabismic amblyopia. Mansouri and his colleagues used a coarse orientation discrimination task (vertical vs. horizontal) to investigate the perception of static second-order stimuli in both anisometropic and strabismic amblyopia. In Simmers and her colleagues' study, a motion direction discrimination task with four different varieties of second-order motion (modulations of either the contrast, flicker, size or orientation of visual noise) were used, but only patients with strabismic amblyopia participated in their study. In the present study, we used a coarse contrast-defined motion direction discrimination task (leftwards vs. rightwards), and only patients with anisometropic amblyopia were employed. Despite these different types of tasks and amblyopia, all these studies found poor perception of second-order gratings in amblyopic eyes, no matter static or moving, suggesting substantial deficits in the early processing of second-order stimuli.

Perception of second-order translational motion has been studied by Simmers and her colleagues [31], [32]. Again, significantly reduced performance has been found in amblyopic eyes. Some of these deficits (‘motion deficits’ as referred to in their studies) have also been indicated to be independent of low-level visibility loss, which seems to be consistent with our findings. However, it should be noted that there are some differences between their findings and ours. With the same method, Simmers and her colleagues also found similar processing deficits for first-order global motion [32], and for both first-order and second-order global orientation integration [31] in amblyopia, indicating that these deficits are not constrained to second-order motion and may be associated with the global processing. In line with this idea, it was found that amblyopic eyes exhibited normal performance levels in similar tasks when the requirements on global processing were decreased [17], [48]. Therefore, it is reasonable to speculate that the processing deficits revealed by Simmers and her colleagues occur at a later processing stage of second-order information, and are different from those for second-order moving gratings, which has been suggested by the present study.

Aaen-Stockdale and his colleagues have evaluated the perception of second-order radial and rotational motion, which are often referred to as optic flow, in amblyopia [33]. Similar to the findings by Simmers and her colleagues described above, they found substantial processing deficits, which were independent of the amblyopic contrast sensitivity deficits, underlying the perception of radial and rotational motion. They further indicated that the deficits in second-order optic flow processing were equivalent to those for first-order stimuli, and were comparable in both eyes, which is clearly different from the deficit pattern found in the present study. Additionally, they found that radial motion deficits were significantly correlated with translational motion deficits, which may suggest that the impairment at the level of global translational motion processing may underlie the radial motion deficits. On this basis, it is clear that the findings of Aaen-Stockdale and his colleagues may suggest a high-level binocular locus underlying the processing deficits of second-order optic flow in amblyopia, which is different from those suggested in the present study.

Despite some differences, a number of previous studies have indicated some deficits of non-amblyopic eyes in the perception of static/moving second-order stimuli. With a detection task, Wong and his colleagues found 2 out of 5 non-amblyopic eyes showed second-order loss relative to the control eyes [29]. Mansouri and his colleagues found that, as a group, eight amblyopes showed reduced contrast sensitivity relative to normal controls in a second-order orientation discrimination task [30]. As for second-order motion, the fellow eye of one amblyopic subject participated in Simmers and her colleagues' study showed poorer performance averaged across four types of second-order stimuli than normal controls [34]. Similar to all these findings, we also find impaired contrast sensitivity of one non-amblyopic eye (S7) for second-order moving gratings and of three eyes (S2, S4 and S7) for static second-order stimuli in the present study. This result adds to the literature that the perception of second-order gratings could be affected by amblyopia for both the amblyopic and the non-amblyopic eyes, supporting the hypothesis proposed by Wong and his colleagues that a binocular mechanism may be involved in the early processing deficits of second-order stimuli in amblyopia.

### Static and moving second-order stimuli

To date, few studies have focused on the processing difference between static and moving second-order stimuli, although the sensitivity difference between second-order form perception and motion perception has been clearly indicated [39], [49]. The proposed systems for the processing of static second-order stimuli [50], [51] share formal properties with the systems for second-order motion processing [23], [52], [53], at least in the initial three processing stages, i.e. filter-rectify-filter. On this basis, the perception of static and moving second-order gratings may have the same characteristics in amblyopic eyes. However, we found that in the present study some amblyopes (S2 and S4) showed impaired contrast sensitivity for static but not for moving second-order gratings, inconsistent with the idea described above. Interestingly, Ellemberg and his colleagues have also found that subjects who experienced early visual deprivation exhibited impaired performance in second-order motion tasks, but not in static tasks [54]. These findings may suggest a dissociation between the processing of static and moving second-order gratings in amblyopia.

Studies on development and aging have also indicated some differences in processing between static and moving second-order gratings. Bertone and his colleagues found a faster developmental rate for perception of moving than of static second-order gratings in school-aged children [55]. Tang and Zhou have also indicated that during adulthood, contrast sensitivity for moving second-order gratings degrades with a much faster rate than for static gratings, although they may have the similar onset age of degradation [45]. These findings support the hypothesis that there may be different neural basis underlying the perception of static and moving second-order stimuli.

Allard and Faubert have shown that there may be two fundamentally different mechanisms underlying the processing of second-order motion: one low-pass and distinct from the mechanisms processing first-order motion and the other common to the mechanisms for first-order motion processing [56]. The former works at all temporal frequencies, whereas the latter only at high frequencies. This might be a possible explanation of the differences in the perception of static (i.e. a temporal frequency of 0) and moving (including many temporal frequencies, from low to high, as in the present study) second-order stimuli described above. Consistent with this idea, it has been indicated that, during adulthood, the sensitivity decline rates were more similar between first-order and second-order moving gratings than between static and moving second-order gratings [45]. This idea has also been supported, indirectly, by studies on perceptual learning in normal adults, which have shown that the effect of training can be transferred, at least partially, from second-order to first-order motion [57]–[59], but not from static second-order to first-order letters [60]. And studies of adaptation, which have shown that cross-over (i.e. from second-order to first-order) adaptation effects occurs, at least partially, for motion [61] but not for static patterns [62], also lend some support to this idea.

### Visual processing in extra-striate cortex in amblyopia

Many studies [23], [50], [63], [64] have suggested that first- and second-order information are separately encoded, in parallel, in the mammalian visual system, and that more processing stages, such as rectification and a second filter, are required for the latter. These additional stages are critical for second-order processing, and are believed to be conducted mainly in extra-striate cortex. This idea is supported by a number of animal electrophysiological studies which have found neurons selective for second-order properties in extra-striate cortex, such as V2 [25], [65], [66] and MT [67], but little in striate cortex [68]. In the present study by eliminating possible influence of low-level processing deficits, we still found poor perception of second-order moving gratings in anisometropic amblyopia, suggesting that the core mechanisms for second-order processing, which may mainly originate from extra-striate cortex, are strongly affected by amblyopia. In other words, our findings support the idea that the processing deficit is not constrained in V1, but also involves large regions of the extra-striate cortex in amblyopia, which has been suggested by a large amount of evidence from animal [13], [21], [22], fMRI [16], [19], [69]–[71] and human psychophysical studies [14], [15], [17], [18], [20], [31]–[33], [72]–[76].

The extra-striate cortex are also involved in the plaid perception. Although some studies [77], [78] have suggested that a number of neurons in primate V1, whose receptive fields are short and wide, could directly encode the plaid stimuli, it has been widely accepted that the plaid patterns are mainly encoded at extra-striate cortex, where a combination of motions of component gratings is implemented [50], [79], [80]. Recently, some studies have reported relatively normal plaid perception in amblyopia. Thompson and his colleagues investigated the plaid coherent perception in 15 patients with strabismic or strabismic-anisometropic amblyopia [81]. Based on the results of three experiments, they found nearly comparable performance between amblyopic, fellow and normal control eyes, and therefore concluded that the neural mechanisms underlying plaid perception are only subtly abnormal in amblyopia. With a motion direction discrimination task, we also found [46] that the loss of contrast sensitivity for moving plaids was statistically equivalent to that for moving component gratings in amblyopic eyes, suggesting the integration of motion information conveyed by component gratings of moving plaids, which is processed at extra-striate cortex, may be intact in amblyopia. All these findings seem to be against the idea described above. However, by using fMRI, another study by Thompson and his colleagues [82] suggested that compared to the normal visual system, the amblyopic visual system may recruit some extra visual areas, such as V3, to support the plaid perception. On this basis, the findings of relatively normal plaid perception in amblyopia do not conflict with the idea that there are substantial processing deficits in extra-striate cortex in amblyopia. Furthermore, it suggests that the visual processing in extra-striate cortex in amblyopia may be more complex than we expected.

### Implications on the treatment of amblyopia

It has been proposed that perceptual learning is a potential therapy for amblyopia. Various visual functions, such as vernier acuity [83], [84], position discrimination in noise [85]–[87], spatial contrast sensitivity [88], [89], visual acuity [88], [90], [91], the perception of first-order motion [92], and binocular vision [93], [94] can be improved through perceptual learning. As for second-order perception, Chung and her colleagues have indicated that it can benefit from training with static letters, both luminance-defined and contrast-defined [95], [96], suggesting substantial plasticity in the visual pathway for processing second-order spatial information. On this basis, it is reasonable to speculate that the poor contrast sensitivity for second-order motion in amblyopia may also be improved with similar training, but with the use of moving stimuli, rather than static ones.

## Conclusion

In this study, we found poor contrast sensitivity for second-order moving gratings in anisometropic amblyopia, which could not be attributed to the impaired first-order input. These results suggest substantial processing deficits for second-order moving gratings, part, if not most, of which might originate from extra-striate cortex. We also found that some amblyopes showed impaired performance in perception of static second-order stimuli but not in that of second-order moving gratings, which may suggest a dissociation between the processing of static second-order stimuli and second-order motion in anisometropic amblyopia.
